# Comparison of clinical outcomes in patients undergoing mitral valve replacement with mechanical or biological substitutes: a 20 years cohort

**DOI:** 10.1186/1471-2261-14-146

**Published:** 2014-10-18

**Authors:** Angela Henrique Silva Ribeiro, Orlando Carlos Belmonte Wender, Adriana Silveira de Almeida, Luciana Eltz Soares, Paulo Dornelles Picon

**Affiliations:** Clinical Medicine of the Federal University of Rio Grande do Sul (UFRGS), Av. Francisco Trein, 596, sala 201, Porto Alegre, RS 91350-200 Brazil; Surgery Department of the Faculty of Medicine, Federal University of Rio Grande do Sul (UFRGS), Cardiovascular Surgery Service for the Hospital de Clínicas de Porto Alegre, Porto Alegre Rio Grande do Sul, Brazil; Cardiology and Cardiovascular Sciences of the Federal University of Rio Grande do Sul (UFRGS), Cardiovascular Surgery, Cardiovascular Surgery of Hospital Nossa Senhora da Conceição, Porto Alegre, Rio Grande do Sul Brazil; Faculty of Medicine of the Federal University of Rio Grande do Sul (UFRGS), National Council for Scientific and Technological Development (CNPq), Porto Alegre, Rio Grande do Sul Brazil; Cardiology by Federal University of Rio Grande do Sul (UFRGS), Internal Medicine, Medical School at UFRGS, Clinical Pharmacology at University of Passo Fundo, Porto Alegre, Rio Grande do Sul Brazil

**Keywords:** Mitral valve surgery, Mitral prosthesis, Bioprostheses, Mechanical prostheses, Predictors, Mortality

## Abstract

**Background:**

The choice of prosthesis for mitral valve replacement still remains controversial. This study assessed mortality, bleeding events and reoperation in patients who underwent mitral valve replacement surgery with biological or mechanical substitutes.

**Methods:**

A total of 352 patients who underwent mitral valve replacement surgery between 1990 and 2008 with 5 to 23 years of follow-up were retrospectively evaluated in a cohort study.

**Results:**

The 5, 10, 15 and 20 year survival rates after surgery using a mechanical substitute were 87.7%, 74.2%, 69.3% and 69.3%, respectively, while after surgery with a biological substitute, they were 87.6%, 71.0%, 64.2% and 56.6%, respectively. There was no significant difference between the two groups (p = 0.38). In the multivariate analysis, the factors associated with death were age, bleeding events and renal failure. The probabilities of remaining free of reoperation at 5, 10, 15 and 20 years after surgery using a mechanical substitute were 94.4%, 92.7%, 92.7% and 92.7%; after surgery with a bioprosthesis, they were 95.9%, 86.4%, 81.2% and 76.5%, respectively (p = 0.073). There was a significantly higher incidence of reoperation for the bioprosthetic valve replacement group (p = 0.008). The probabilities of remaining free of bleeding events at 5, 10, 15 and 20 years after surgery using a mechanical substitute were 95.0%, 91.0%, 89.6% and 89.6%, respectively, while after surgery with a bioprosthesis, they were 96.9%, 94.0%, 94.0% and 94.0%, (p = 0.267).

**Conclusions:**

The authors concluded that: 1) mortality during follow-up was statistically similar for both groups; 2) there was a greater tendency to reoperation in the bioprosthesis group; 3) the probability of remaining free from reoperation remained unchanged after 10 years’ follow-up for patients with mechanical substitute valves; 4) the probability of remaining fee from bleeding events remained unchanged after 10 years’ follow-up for patients given bioprostheses; 5) the baseline characteristics of patients were the greatest determinants of later mortality after surgery; 6) the type of prosthesis was not an independent predictive factor of any of the outcomes tested in the multivariate analysis.

## Background

Mitral valve replacement is a surgical procedure employed when a valve is so severely compromised that preservation is not viable, ruling out reparative surgery, and it is also recommended for initial phase mitral insufficiency in young patients, irrespective of symptoms, according to the American Heart Association (ACC/AHA) [1].

As the longevity of populations steadily extends, the high costs inherent to potential high complexity interventions merit due attention. In Brazil, life expectancy increased by 11% between 1980 and 2000, according to the Brazilian Institute of Geography and Statistics (IGBE - *Instituto Brasileiro de Geografia e Estatística*) and by 2050 there will be 226 elderly people over 60 years for every 100 children and adolescents [2]. It has been shown that heart surgery can be effective in patients in their seventies and eighties, but the cost of operating these patients can be up to 35% higher [3].

Figures provided by DATASUS, the IT department of the Brazilian National Health Service (SUS - *Sistema Único de Saúde*) [4], show that implantation of prosthetic valves accounted for 16.4% of high complexity cardiovascular surgery conducted in Brazil between January 2008 and July 2013, with 40,506 operations to implant valves, compared with 3,683 valvuloplasty operations during the same period. Twenty-five percent of Brazilian government spending on health services is spent on cardiovascular care [5].

More than 30 years after the introduction of modern prosthetic valves, the choice of whether to use a biological or a mechanical valve in the mitral position is still the subject of debate [6, 7]. This is because there is no ideal substitute [8–11] that offers long-term durability, without the need for oral anticoagulants, no increased risk of thromboembolism and a functional mechanism similar to the native mitral valve [10]. This decision becomes an even greater challenge when patients have coexisting conditions, such as advanced age, congestive heart failure, coronary artery disease, lung disease or renal failure. The increase in life expectancy and in comorbidities among patients needing valve replacement means that choosing the most effective treatment (valvuloplasty, mechanical prosthesis or bioprosthesis) demands consideration of additional factors [12].

The objective of this study was to investigate mortality, bleeding events and reoperation among patients who underwent surgery for mitral valve replacement with a biological or mechanical prosthesis in a tertiary hospital that is a heart surgery referral center for the South of Brazil.

## Methods

The study design was a historical, observational cohort study.

### Sample

**Figure 1:**
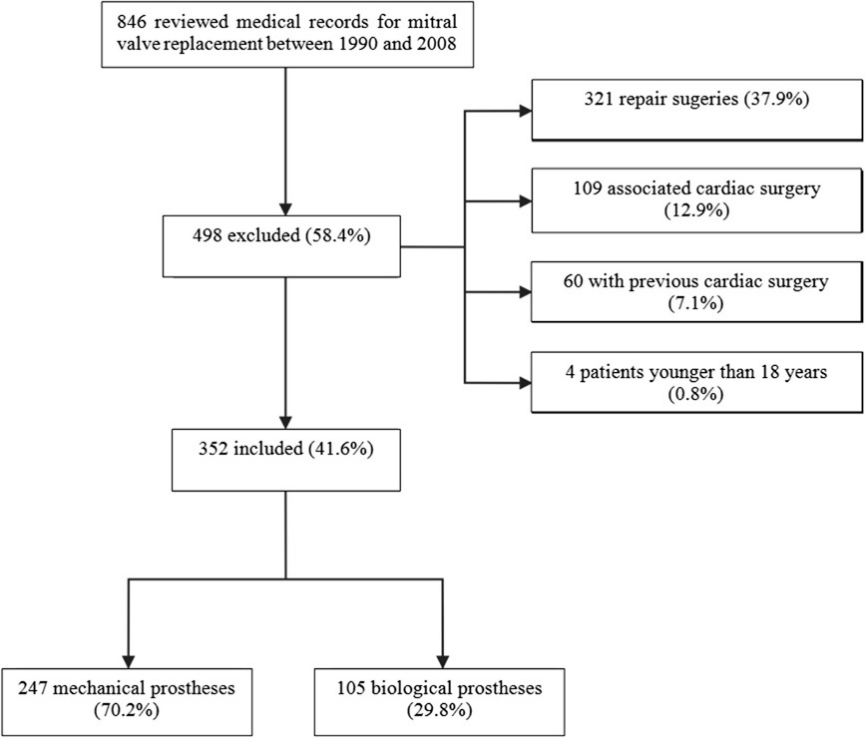
**Flowchart of patients.** This flowchart shows the inclusion and exclusion criteria of the studied subjects.

Data were obtained from the archive service at the Hospital de Clínicas de Porto Alegre (HCPA), Rio Grande do Sul, Brazil, from the medical records of 846 inpatients over the age of 18 who had undergone surgery in that hospital for mitral valve replacement between 1 January 1990 and 31 December 2008 and had been followed-up for outcomes up to June 2013. Cases with mitral valvuloplasty (n = 321) additional heart surgery of other types (n = 109), previous heart surgery (n = 60) and patients under the age of 18 (n = 4) were all excluded, leaving a cohort of 352 patients (Figure 1). The PEPI (Programs for Epidemiologists) version 4.0 software package was used to calculate the sample size needed to detect an effect size (difference between groups) with relation to mortality of 15% between prosthesis types while maintaining a statistical power of 80% and a 5% significance level. The effect size was estimated from data published by Hammermeister et al. [8, 13, 14]. With these parameters, the minimum sample size was estimated at 314 cases.

The principal objective was to compare mortality between patients given mechanical or biological replacement valves. Secondary objectives were: 1) to compare the probability of survival free from reoperation and bleeding events across groups; and 2) to analyze predictors of death, reoperation and bleeding events.

Clinical and surgical features of the cases were harvested from information on patient records. The data thus collected were evaluated by at least two authors independently. The team's performance was subjected to quality control in the form of double input data entry with crosschecking. The study methodology was based on the Strengthening the Reporting of Observational Studies in Epidemiology (STROBE) [15].

Records of deaths were obtained from the Rio Grande do Sul State Health Department healthcare information service in Porto Alegre.

Complications related to prostheses were recorded in accordance with the Guidelines for Reporting Mortality and Morbidity After Cardiac Valve Interventions [16].

All operations were conducted under extracorporeal circulation, with moderate hypothermia (32°) and cardiac arrest, following the standardized technique adopted by the Cardiovascular Surgery Department at the Hospital de Clínicas de Porto Alegre, including anesthetic procedures. All of the mechanical prostheses employed were bileaflet valves and all of the biological prostheses implanted were provided by the SUS. After surgery, all patients were transferred to the postoperative heart surgery ICU on mechanical ventilation. Maximum duration of follow-up was 23 years, with a mean of 9.2 ± 4.8 years and median of 8.9 years.

### Ethical considerations

The research project was approved in advance by the Medical Research Ethics Committee at the Hospital de Clínicas de Porto Alegre, under protocol number 11–0497, in order to obtain permission to conduct the study in that hospital, with financial support from the hospital’s research funding agency, the *Fundo de Incentivo à Pesquisa e Eventos* (FIPE/HCPA).

Patient confidentiality and anonymity were guaranteed. Use of the data collected for this study was restricted to the objectives of this project.

### Definitions

The definitions listed below are all taken from the Guidelines for Reporting Mortality and Morbidity After Cardiac Valve Interventions [15].

The total number of deaths includes all deaths, from whatever causes, of patients who had had mitral valve surgery.

Early mortality is defined as all deaths within 30 days of surgery, irrespective of the patient’s location.

Hospital mortality is any death after surgery while still in hospital.

Valve-related mortality is defined as any death caused by structural deterioration, nonstructural dysfunction, thrombosis, embolism, bleeding events, endocarditis, or death related to reoperation of a previously operated valve. Deaths caused by heart failure in patients with advanced myocardial disease and no valve dysfunction are not included in this category.

Cardiac deaths are all deaths resulting from cardiac causes, including deaths related and unrelated to valves or prostheses. This category includes deaths from congestive heart failure, acute myocardial infarction and documented arrhythmias, among others.

Sudden, unexplained and unexpected death are deaths from unknown causes and their relationship with the operated valve is also unknown. This item is a separate category from valve-related mortality, to cover cases when the cause cannot be determined from clinical or necropsy findings.

Reoperation is when a previously operated valve is repaired, altered, adjusted or replaced, according to the Guidelines for Reporting Morbidity and Mortality after Cardiac Valvular Interventions [16].

A bleeding event is defined as any episode of major internal or external bleeding that causes death, hospita-lization, or permanent injury, such as a cerebral vascular accident or loss of vision or bleeding requiring blood transfusions.

### Statistical analysis

Quantitative variables were described using means and standard deviations, where distribution was symmetrical, or medians and interquartile range, in cases of asymmetrical distribution, and qualitative variables were expressed as absolute and relative frequencies. Groups were compared using Student’s *t* test for independent samples (symmetrical distribution) or the Mann–Whitney test (asymmetrical distribution) for quantitative variables, and Pearson’s chi-square or Fisher’s exact test for qualitative variables (rates and proportions).

Survival rates and probabilities of reoperation and of bleeding events were assessed using Kaplan-Meier curves. The log-rank chi-square test was used to compare curves across groups.

Cox's proportional risk model was employed to control for confounding factors. Hazard ratios and 95% confidence intervals were used to measure the effect. For all models, the criterion for a variable to be entered was a p value below 0.20 on bivariate analysis, with the exception of type of prostheses, which was included in all models since it was the principal factor under study.

The significance level was set at 5% and data were analyzed using the program SPSS (Statistical Package for the Social Sciences) version 17.0.

## Results

As illustrated in Figure 1, 247 (70.2%) of the patients were given mechanical prosthesis and 105 (29.8%) had a bioprosthesis implanted (p ≤ 0.001).

Figure 1 lists the characteristics of the patients selected for the study sample.

Patients who were fitted with mechanical prostheses were younger, had higher body mass index and had a higher proportion of sinus rhythm on electrocardiogram (ECG) and of elective surgery than patients given biological replacement valves (P < 0.05). The remaining preoperative characteristics were similar across both groups (Table 1).

**Table 1:** **Sample characterization**

Variable	Sample (n = 352)	Mechanical valve (n = 247)	Biological valve (n = 105)	p
Mean age ± SD	52.3 ± 13.4	50.8 ± 12.5	55.8 ± 14.9	0.003
Age group - n (%)				
≤ 50 years	161 (45.7)	118 (47.8)	43 (41.0)	< 0.001
51 - 60 years	99 (28.1)	77 (31.2)*	22 (21.0)	
61 - 70 years	58 (16.5)	38 (15.4)	20 (19.0)	
≥ 71 years	34 (9.7)	14 (5.7)	20 (19.0)*	
Gender - n (%)				
Male	165 (46.9)	116 (47.0)	49 (46.7)	1.000
Female	187 (53.1)	131 (53.0)	56 (53.3)	
BMI (Kg/m^2^) - Mean ± SD	24.4 ± 4.3	24.8 ± 4.5	23.4 ± 3.8	0.003
Obesity** - n (%)	33 (9.4)	27 (10.9)	6 (5.7)	0.181
Morbid obesity*** - n (%)	1 (0.3)	1 (0.4)	0 (0.0)	1.000
Functional class (NYHA) - n (%)				
I-II	203 (57.7)	143 (57.9)	60 (57.1)	0.990
III-IV	149 (42.3)	104 (42.1)	45 (42.9)	
Pathology – n (%)				0.719
Failure	155 (44.0)	104 (42.1)	51 (48.6)	
Stenosis	83 (23.6)	61 (24.7)	22 (21.0)	
DI with predominant stenosis	84 (23.9)	60 (24.3)	24 (22.9)	
DI with predominant failure	30 (8.5)	22 (8.9)	8 (7.6)	
Rhythm ECG – n (%)				
Sinus	147 (42.0)	114 (46.3)*	33 (31.7)	
Atrial fibrillation	198 (56.6)	129 (52.4)	69 (66.3)*	0.039
Others	5 (1.4)	3 (1.2)	2 (1.9)	
Chronic atrial fibrillation - n (%)	169 (48.0)	112 (45.3)	57 (54.3)	0.156
Diabetes mellitus - n (%)	25 (7.1)	18 (7.3)	7 (6.7)	1.000
COPD - n (%)	56 (15.9)	39 (15.8)	17 (16.2)	1.000
Stroke - n (%)	24 (6.8)	17 (6.9)	7 (6.7)	1.000
SAH - n (%)	144 (40.9)	105 (42.5)	39 (37.1)	0.413
Creatinine > 2 mg/dl – n (%)	17 (4.8)	9 (3.6)	8 (7.6)	0.187
Dialysis - n (%)	3 (0.9)	1 (0.4)	2 (1.9)	0.213
Emergency surgery - n (%)	22 (6.3)	10 (4.0)	12 (11.4)	0.017
Endocarditis - n (%)	36 (10.2)	22 (8.9)	14 (13.3)	0.288
Rheumatic fever - n (%)	153 (43.5)	112 (45.3)	41 (39.0)	0.331

SD, standard deviation; BMI, body mass index; NYHA, New York Heart Association class; DI, double injury, ECG, electrocardiogram; COPD, chronic obstructive pulmonary disease; SAH, systemic arterial hypertension.

*Statistically significant association by the adjusted residuals test at 5% significance.

**BMI ≥ 30 kg/m^2^; ***BMI ≥ 40 kg/m^2^.

The patients who underwent bioprosthesis implantation experienced a significantly longer surgical time (<0.001) (Table 2).

**Table 2:** **Surgery**

Variables	n	Total sample	Mechanical valve (n = 247)	Biological valve (n = 105)	p
Total surgical time (min) - mean ± SD	348	183.2 ± 61.2	175.5 ± 60.0*	202.5 ± 60.5	< 0.001
ECC time (min) - mean ± SD	352	76.5 ± 33.2	75.4 ± 34.7	79.2 ± 29.4	0.322
ECC > 120 min - n (%)	352	35 (9.9)	20 (8.1)	15 (14.3)	0.114
Ischemia time (min) - mean ± SD	350	57.6 ± 25.4	57.2 ± 24.7	58.4 ± 27.0**	0.691

SD, standard deviation; ECC, extracorporeal circulation; min, minutes.

*n = 243; **n = 103.

In-hospital outcomes broke down as follows: bioprosthesis patients spent longer in hospital (p < 0.001), spent longer on mechanical ventilation (p < 0.001) and spent longer periods in the intensive care unit (ICU) (p = 0.009), when compared with mechanical prosthesis patients, as shown in Table 3. There were no statistically significant differences between groups for prolonged mechanical ventilation (more than 5 days), acute myocardial infarction, cerebral vascular accident, arrhythmia requiring cardioversion or defibrillation, dialysis, reoperation due to bleeding, cardiac tamponade or total, permanent atrioventricular block (p > 0.05). Time on extracorporeal circulation and with the aorta clamped were similar for both groups (p > 0.05).

**Table 3:** **Postoperative outcomes**

Variable	Sample (n = 352)	Mechanical prosthesis (n = 247)	Biological prosthesis (n = 105)	p
Hospitalization time (days) - median (P25–P75)	13 (10–20)	12 (9–18)	17 (13–27)	< 0.001
ICU time (days) - median (P25–P75)	3 (2.8–4)	3 (2.8–3.8)	3.3 (2.9–4.2)	0.009
PO hospitalization time (days) - median (P25–P75)	8 (7–11)	8 (7–10)	10 (8–14)	< 0.001
Mechanical ventilation time (h) - median (P25–P75)	15 (9–20)	14 (8–20)	17 (12–24)	< 0.001
Mechanical ventilation > 5 days - n (%)	7 (2.0)	4 (1.6)	3 (2.9)	0.431
AMI - n (%)	3 (0.9)	2 (0.8)	1 (1.0)	1.000
Stroke - n (%)	7 (2.0)	5 (2.0)	2 (1.9)	1.000
Arrhythmia requiring cardioversion - n (%)	13 (3.7)	7 (2.8)	6 (5.7)	0.219
Dialysis	2 (0.6)	0 (0.0)	2 (1.9)	0.088
Reoperation due to bleeding - n (%)	15 (4.3)	10 (4.0)	5 (4.8)	0.776
Cardiac tamponade - n (%)	10 (2.8)	7 (2.8)	3 (2.9)	1.000
Permanent TAVB	4 (1.1)	2 (0.8)	2 (1.9)	0.586

P25 = 25th Percentile; P75 = 75th Percentile; ICU, intensive care unit; PO, postoperative; AMI, acute myocardial infarction; TAVB, total atrioventricular block.

### Survival data

**Figure 2:**
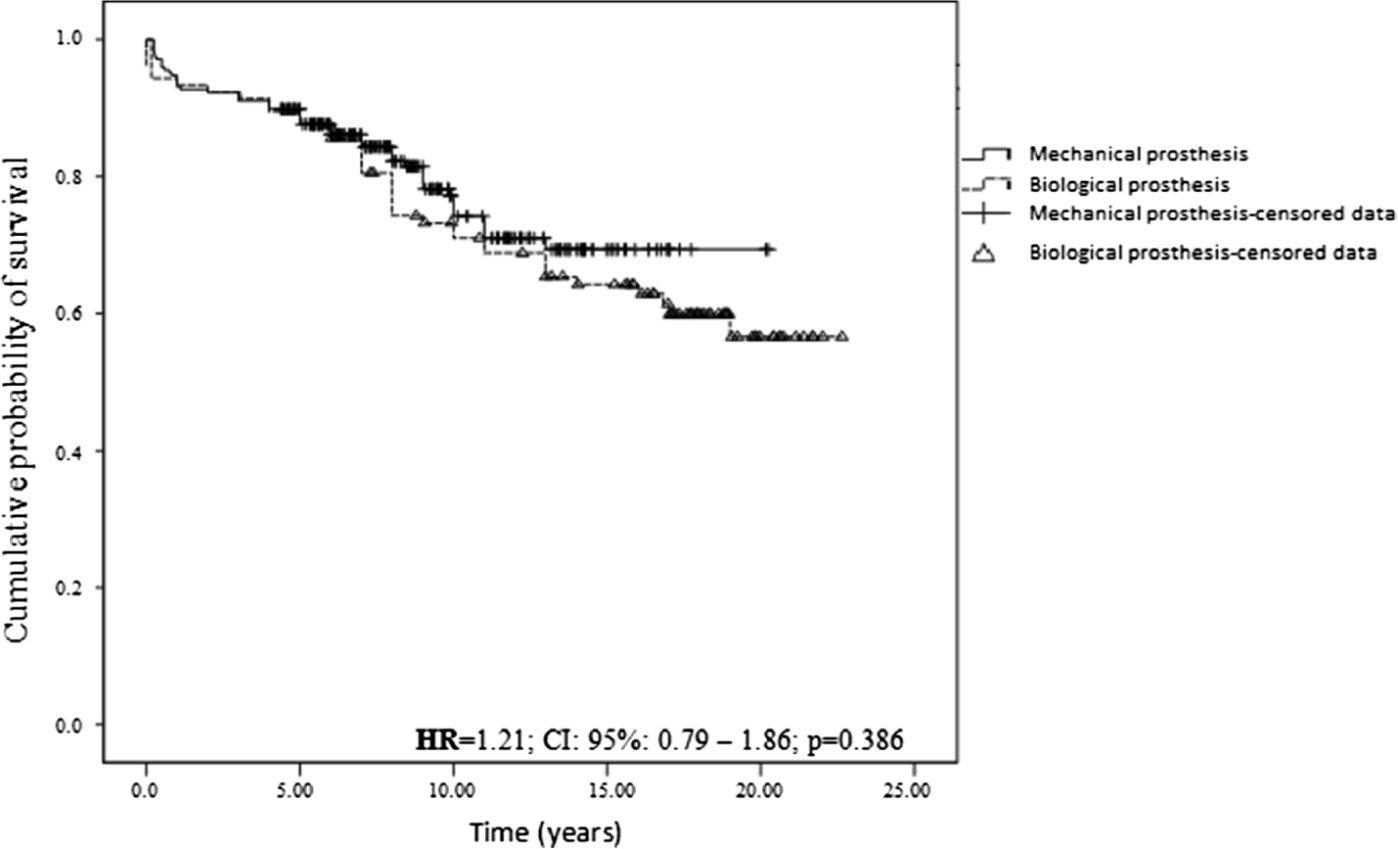
**Cumulative probability to survival based on the type of valve prosthesis.** Kaplan-Meier curve to assess the cumulative probability to survival based on the type on the valve prosthesis.

The long-term survival rates of the patients in this study are illustrated in Figure 2. The 5, 10, 15 and 20 year survival rates after valve replacement surgery by a mechanical substitute were 87.7% (CI: 83.6-91.8), 74.2% (CI: 67.3-81.1), 69.3% (CI: 61.3-77.3) and 69.3% (CI: 61.3-77.3), respectively. For the patients who received a biological substitute, the survival rates were 87.6% (CI: 81.3-93.9), 71.0% (CI: 62.0-80.0), 64.2% (CI: 54.6-73.8) and 56.6% (CI: 45.0-68.2), respectively. Thus, there was no significant difference in the survival of the patients between the two groups (p = 0.386) throughout the follow-up period.

The multivariate Cox regression analysis demonstrated no association between the type of valve and death (p = 0.855), as shown in Table 4. The factors statistically associated with the death outcome were age over 60 years (61–70: Harzard ratio [HR] = 1.98; 95% Confidence Interval [CI]: 1.11–3.56; ≥71 years: HR = 2.96; 95% CI: 1.54–5.71), renal failure (HR = 4.41; 95% CI: 2.36–8.25) and bleeding events (HR = 3.13; 95% CI: 1.68–5.80).

**Table 4:** **Predictors of death by Cox regression analysis**

Variable	HR (95% CI)	p
Mechanical prosthesis	0.96 (0.61-1.50)	0.855
**Renal failure**	**4.41 (2.36-8.25)**	**< 0.001**
**Bleeding events**	**3.13 (1.68-5.80)**	**< 0.001**
**Age group**		
≤ 50 years	1.0	
51–60 years	1.39 (0.82-2.38)	0.224
**61–70 years**	**1.98 (1.11-3.56)**	**0.022**
**≥ 71 years**	**2.96 (1.54-5.71)**	**0.001**
Mitral regurgitation	1.49 (0.97-2.29)	0.069
SAH	1.45 (0.95-2.23)	0.088
Chronic atrial fibrillation	1.46 (0.91-2.33)	0.114
Emergency surgery	1.69 (0.82-3.47)	0.153
Diabetes mellitus	1.62 (0.82-3.19)	0.163
Ischemia time	1.01 (0.99-1.01)	0.180
ICU time (h)	1.00 (0.99-1.00)	0.492
Hospitalization time	1.01 (0.99-1.02)	0.501
MV time (h)	1.00 (1.00-1.01)	0.589
Diameter	1.03 (0.92-1.15)	0.654
CHF class III and IV	1.10 (0.70-1.75)	0.675
Postoperative stroke	1.36 (0.31-5.99)	0.681
COPD	0.91 (0.48-1.70)	0.759
ECC time > 120 min	1.11 (0.40-3.10)	0.843
Rheumatic fever	1.02 (0.60-1.71)	0.949

HR, hazard ratio; 95% CI = 95% confidence interval; BMI, body mass index;

ECC, extracorporeal circulation; ICU, intensive care unit; MV, mechanical ventilation; CHF, congestive heart failure; AMI, acute myocardial infarction; COPD, chronic obstructive pulmonary disease; SAH, systemic arterial hypertension.

Significant results (p < 0.005) are highlighted in bold.

As the groups differed significantly for age, body mass index (BMI, Kg/m^2^), electrocardiogram (ECG) rhythm and emergency surgery, a propensity score was added to the model to minimize confounding biases into the study. Even after adjusting for propensity score, there was no modification of the results obtained by Cox regression. The risk of death for mechanical prosthesis was very similar when adjusted for propensity score, with HR = 0.97 (95% CI: 0.62-1.53, p = 0.93).

A significantly higher incidence of reoperation for valve replacement (p = 0.008) and death (p = 0.003) was found in patients who underwent biological valve replacement (Table 5).

**Table 5:** **Outcomes in the cohort during the follow-up period of up to 23 years**

Variable	Sample (n = 352) n (%)	Mechanical prosthesis (n = 247) n (%)	Biological prosthesis (n = 105) n (%)	p
Reoperation for valve replacement	33 (9.4)	16 (6.5)	17 (16.2)	0.008
Bleeding events	23 (6.5)	18 (7.3)	5 (4.8)	0.521
Thromboembolic events	7 (2.0)	5 (2.0)	2 (1.9)	1.000
Total number of deaths	91 (25.9)	52 (21.1)	39 (37.1)	0.003
Perioperative death	21 (6.0)	10 (4.0)	11 (10.5)	0.037
ICU*	9 (2.6)	4 (1.6)	5 (4.8)	0.133
Hospital*	11 (3.1)	5 (2.0)	6 (5.7)	0.092
Others**	70 (19.9)	42 (17.0)	28 (26.7)	0.053

ICU, intensive care unit; *none exceeded the perioperative period; **deaths after 30 days of hospitalization.

A significantly higher incidence of reoperation for valve replacement (p = 0.008) and death (p = 0.003) was found in patients who underwent biological valve replacement (Table 5).

There was no significant difference between the groups regarding the cause of death (p = 1.000). The most frequent cause was non-cardiac (39.6%), followed by prosthesis-related causes (35.2%), as shown in Table 6.

**Figure 3:**
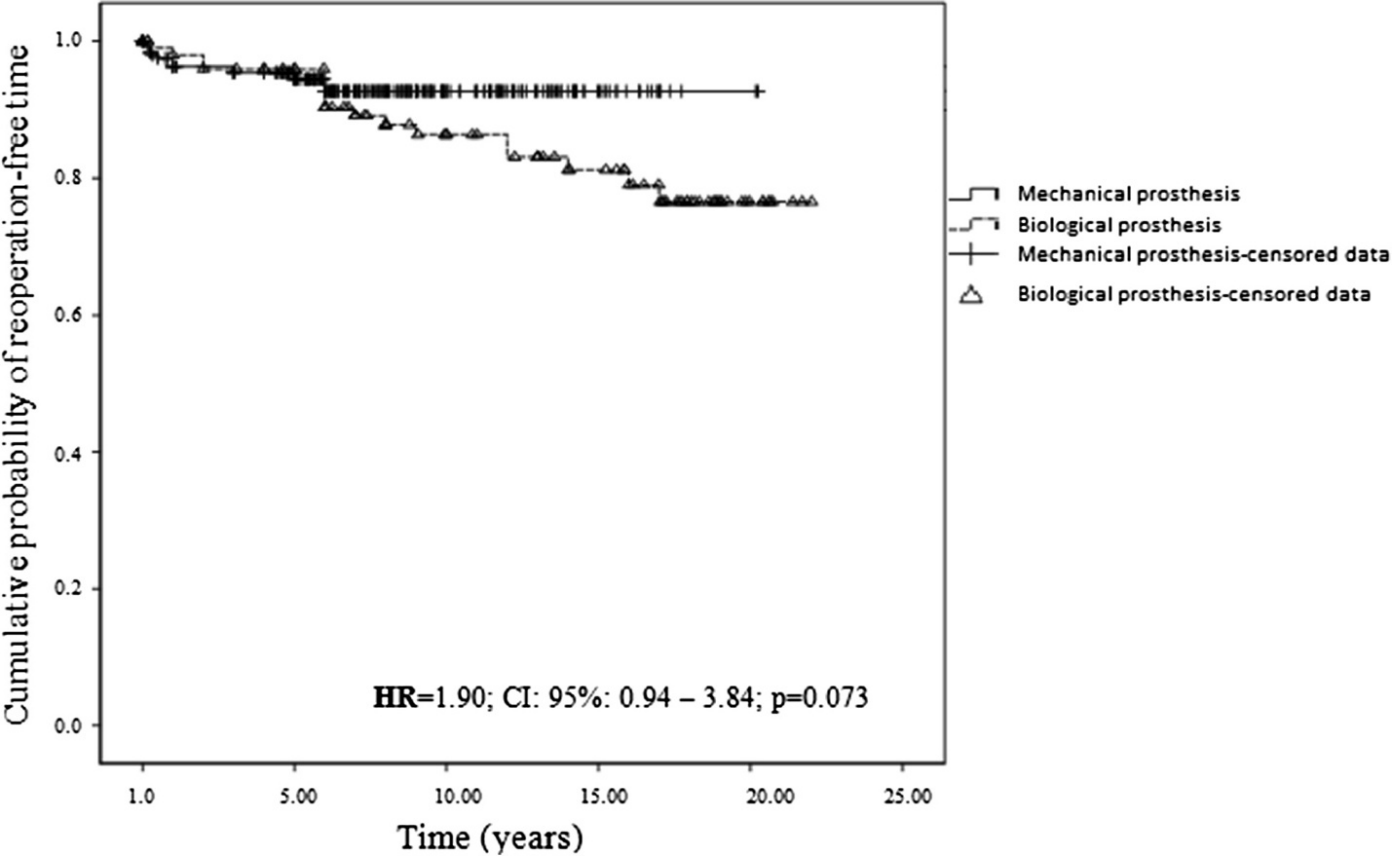
**Cumulative probability of reoperation.** Kaplan-Meier curve to assess the cumulative probability of reoperation-free time according to the type of valve prosthesis.

Figure 3 illustrates the probability of long-term survival free from reoperation for the study patients. The probabilities of survival free from reoperation at 5, 10, 15 and 20 years after surgery for valve replacement with a mechanical prosthesis were 94.4% (CI: 91.5-97.3), 92.7% (CI: 89.2-96.2), 92.7% (CI: 89.2-96.2) and 92.7% (CI: 89.2-96.2) respectively and the figures for biological valves were 95.9% (CI: 92.0-99.8), 86.4% (CI: 79.1-93.7), 81.2% (CI: 72.4-90.0) and 76.5% (CI: 66.1-86.9) respectively. Patients with biological replacement valves tended to have a greater probability of reoperation, particularly after the first 10 years’ follow-up (p = 0.073).

**Table 6:** **Causes of death**

Cause of death *	Sample (n = 91) n (%)	Mechanical prosthesis (n = 52) n (%)	Biological prosthesis (n = 39) n (%)	p
Prosthesis-related	32 (35.2)	18 (34.6)	14 (35.9)	1.000
Others	59 (64.8)	34 (65.4)	25 (64.1)	1.000
Cardiac	21 (23.1)	12 (23.1)	9 (23.1)	1.000
Non-cardiac	36 (39.6)	21 (40.4)	15 (38.5)	1.000
Sudden or unexplained	2 (2.2)	1 (1.9)	1 (2.6)	1.000

*Deaths represent 25.9% (n = 91) of the samples.

As shown in Table 7, after adjustment by Cox multivariate regression, type of valve continued not to exhibit any association with reoperation (p = 0.075). Factors that did remain statistically associated with reoperation after multivariate analysis were male sex (HR = 2.50; 95% CI: 1.20-5.35), diameter of prosthesis (HR = 0.82; 95% CI: 0.69-0.97); and endocarditis (HR = 2.44; 95% CI: 1.04-5.70).

**Table 7:** **Predictors of reoperation by Cox regression analysis**

Variable	HR (95% CI)	p
Biological prosthesis	1.89 (0.94-3.82)	0.075
**Male gender**	**2.54 (1.20-5.35)**	**0.015**
**Valve prosthesis diameter**	**0.82 (0.69-0.97)**	**0.019**
**Endocarditis**	**2.44 (1.04-5.70)**	**0.040**
Arrhythmia	2.92 (0.86-9.89)	0.085
Emergency surgery	1.98 (0.71-5.56)	0.192
RF	1.12 (0.24-5.29)	0.885

HR: hazard ratio; 95% CI = 95% confidence interval; RF = renal failure.

Significant results (p < 0.005) are highlighted in bold.

After adjustment by propensity score, the use of biological prostheses showed an HR = 1.68 (95% CI: 0.82-3.45, p = 0.155), and did not modify the results obtained by Cox regression.

**Figure 4:**
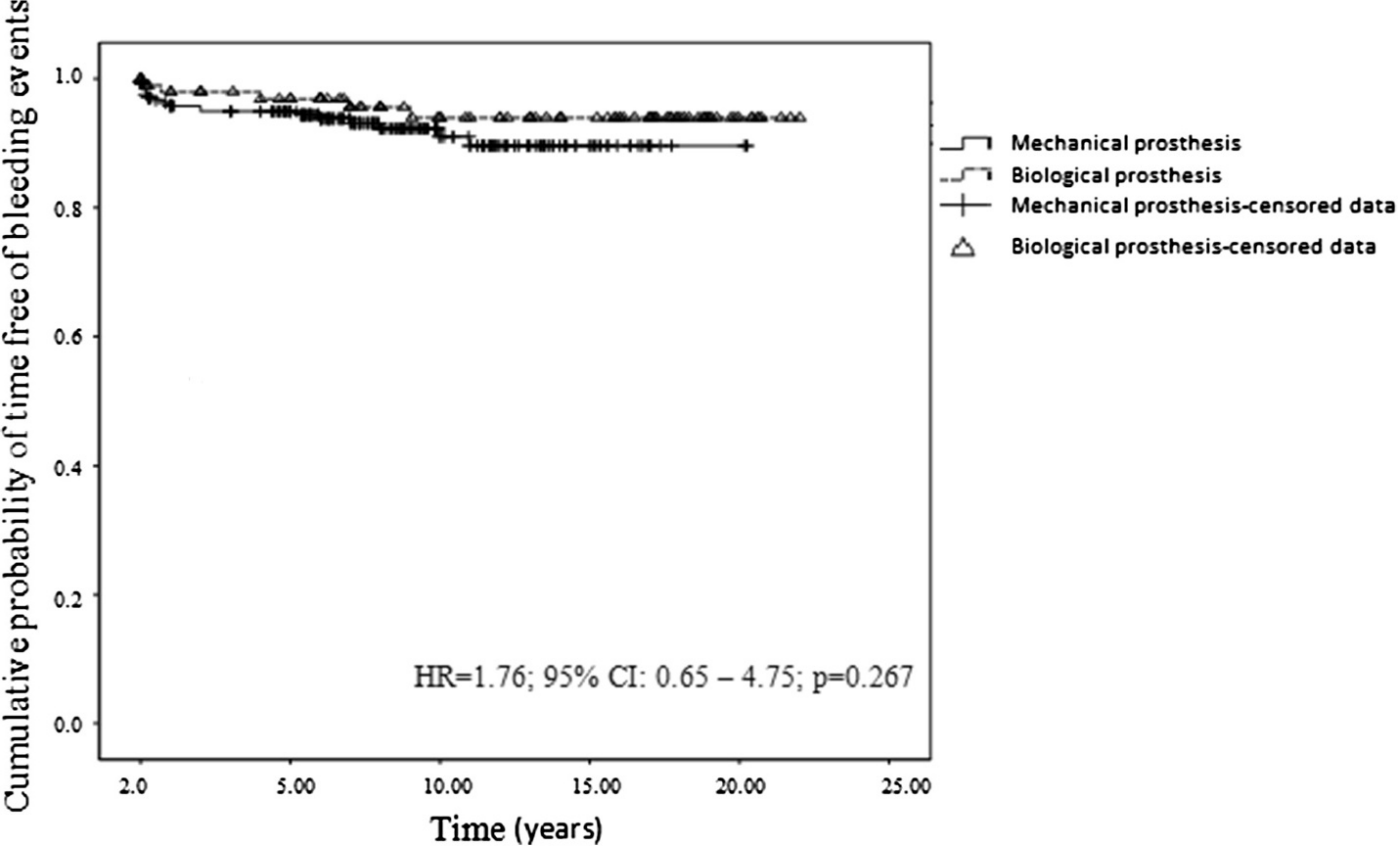
**Cumulative time free of bleeding events.** Kaplan-Meier curve to assess the cumulative of time free of bleeding events according to the type of valve prosthesis.

Figure 4 illustrates the probability of long-term survival free from bleeding events, by type of prosthesis. The probabilities of survival free from bleeding events at 5, 10, 15 and 20 years after surgery for valve replacement with a mechanical prosthesis were 95.0% (CI: 92.3-97.7), 91.0% (CI: 86.5-95.5), 89.6% (CI: 84.5-94.7) and 89.6% (CI: 84.5-94.7) respectively and for patients with biological replacements the figures were 96.9% (CI: 93.6-100), 94.0% (CI: 88.7-99.3), 94.0% (CI: 88.7-99.3) and 94.0 (CI: 88.7-99.3) respectively. There was no statistically significant difference between the 2 groups (p = 0.267).

As shown in Table 8, after adjustment by Cox multivariate regression, type of valve continued not to exhibit any association with the incidence of bleeding events (p = 0.213). Factors that did remain statistically associated with occurrence of bleeding events after multivariate analysis were body mass index ≥ 30 kg/m^2^ (HR = 4.41; 95% CI: 1.66-11.8), chronic obstructive pulmonary disease (HR = 2.87; 95% CI: 1.19-6.91), more than 5 days on mechanical ventilation in the ICU (HR = 5.34; 95% CI: 1.13-25.4) and mitral insufficiency (HR = 2.56; 95% CI: 1.05-6.25).

**Table 8:** **Predictors of bleeding events by Cox regression analysis**

Variable	HR (95% CI)	P
Mechanical prosthesis	1.90 (0.69–5.18)	0.213
**BMI ≥ 30 kg/m** ^**2**^	**4.41 (1.66–11.8)**	**0.003**
**COPD**	**2.87 (1.19–6.91)**	**0.019**
**MV > 5 days**	**5.34 (1.13–25.4)**	**0.035**
**Mitral regurgitation**	**2.56 (1.05–6.25)**	**0.040**
SAH	2.31 (0.96–5.56)	0.062
Ischemia time	1.01 (0.99–1.02)	0.131
Diabetes mellitus	1.74 (0.48–6.36)	0.404
Hospitalization time	1.01 (0.98–1.03)	0.740
Rheumatic fever	1.15 (0.41–3.27)	0.790
RF	1.28 (0.14–11.4)	0.828

HR: hazard ratio; 95% CI = 95% confidence interval; BMI = body mass index; SAH = systemic arterial hypertension; COPD = chronic obstructive pulmonary disease; MV = mechanical ventilation; RF = renal failure.

Significant results (p<0.005) are highlighted in bold.

Considering the presence of bleeding events stratified by age groups, no significant difference were found between groups in years: <60, 60–65, 65–70, 70–75, 75–80, and >80, with p = 0.895, being <60 = 6%, 60-65 = 8.3%, 65-70 = 10.3%, 70-75 = 7.7%, 75-80 = 0.0, >80 = 0. When adjusted for propensity score, no significant change was obtained in the values of the risk measure HR = 2.3 (95% CI: 0.76-5.96, p = 0.149).

## Discussion

### Mortality

The actuarial mortality rate observed in this cohort was 25.9%, and there was no difference between the groups that received the mechanical and biological prostheses throughout the follow-up time (p = 0.386) (Figure 2). This result may have occurred due to the increased risk related to anticoagulation in patients who received the mechanical prostheses being partially offset by the increased risk of reoperation in patients who received the biological prostheses.

Hammermeister et al. [8, 13, 14] observed even higher numbers of deaths in a prospective randomized clinical trial comparing porcine prostheses with mechanical prostheses in 181 patients subjected to mitral valve replacement over an 18 year follow-up period, reporting total mortality of 30%, with 22% related to mechanical prostheses and 16% related to bioprostheses. Five year survival was 58 ± 6% and 70 ± 5% for patients with mechanical and biological prostheses respectively. The elevated number of deaths was probably the result of the fact that these implants were fitted between 1970 and 1980. Many of the deaths of bioprostheses patients happened more than 10 years after surgery and can be attributed to primary prosthesis dysfunction, with or without reintervention. The authors concluded that survival rates were similar for bioprostheses and mechanical prosthesis patients over a mean of eight years.

Our findings are also in agreement with survival results of a randomized clinical trial conducted in Edinburgh by Oxeham and colleagues [17], who compared the outcomes of 261 patients who had had mitral valves replaced with either a mechanical (n = 129) or porcine prosthesis between 1975 and 1979 with a 20-year follow-up period. They found that there was an advantage in terms of survival from 10 years onwards for mechanical prosthesis patients, but that this advantage disappeared by 20 years' follow-up (p < 0.0001). In a similar vein to this study, these authors found that survival rates at 10 and 20 years after valve replacement surgery to fit a mechanical prostheses were 52.7% and 22.4% while for biological prostheses they were 46.5% and 18.4% respectively, which did not attain statistical significance (p = 0.41).

Advanced age has also been identified as a predictive factor in other cohorts that have been studied, including a prospective multicenter cohort study conducted from January to December of 2001 at eight hospitals in the north of the United States. They reviewed 3150 cases of mitral valve surgery including 1688 patients, with a mean age of 66.7, who were given replacement valves (53.6%), observing 12% mortality [18]. In addition to age, a further nine variables were also associated with death after mitral surgery: female sex, diabetes, coronary disease, prior cerebral vascular accident, elevated creatinine (≥1.3 mg/dl), New York Heart Association (NYHA) class IV, heart failure, valve replacement rather than valvuloplasty and emergency surgery.

Likewise, Ruel et al. [19] compared mortality rates for 214 patients younger than 60 years who had surgery for mitral valve replacement with mechanical or biological substitutes between 1969 and 2004, with up to a 35 year postoperative follow-up period (mean survival 24 ± 3.1 years). There was no difference in survival between groups, with rates of 51.4 ± 4.4% and 33.8 ± 5.3% for 20 and 25 years respectively after bioprosthesis implantation and 43.2 ± 5.7% and 40.8 ± 5.9% respectively for mechanical valves. Age group, as observed in this study, was also predictor factor for death.

In a 20-year follow-up cohort study, Khan et al. [20] compared outcomes for 513 patients with mechanical mitral prostheses with 402 given bioprostheses. Mechanical prostheses were preferred for younger patients (p = 0.0001). The same study found no significant difference in survival between prosthesis groups using multivariate analysis. Advanced age was once more a predictor of mortality, as in the cohort described here.

Jamieson et al. [21] found that freedom from prosthesis-related mortality rates were only better than rates for biological replacements in the age group from 51 to 60, with 75.4 ± 8.3% survival for bioprostheses and 87.5 ± 8.7% for mechanical valves, while for patients over 70 rates were similar. The predictors factors for mortality were age, male sex, bioprosthesis, diameter of prosthesis and concurrent revascularization surgery. In the present study, type of prosthesis was not a significant predictor factor of death, with p = 0.461.

After multivariate analysis, Kim and colleagues [22] also identified age group as an independent risk factor for death, echoing Kulik et al. [23], Khan et al. [20], Yau et al. [24] and Jamienson et al. [25]. Some studies attempted to perform risk stratification in heart surgery; however, there are several variables that affect such classification [25–27], such as pulmonary hypertension, functional class, emergency surgery, type of prosthesis, atrial fibrillation, multiple surgeries, renal failure, peripheral vascular disease, non-rheumatic diseases, small body surface and prosthesis-patient mismatch.

### Bleeding events

In the sample described here, 6.5% of cases involved major bleeding events (n = 23) and the majority of these were associated with mechanical prostheses (p = 0.521), with a statistically significant difference. This may be due to the low number of events, to well-controlled anticoagulation of patients given mechanical replacement valves or to anticoagulation prescribed for other indications over the course of the follow-up period.

Some studies associated mechanical prostheses with bleeding events, as an independent risk factor, due to the need for anticoagulation. The study conducted by Khan and colleagues [20] reported a 2.1% bleeding event rate for patients with mitral mechanical prostheses, compared with 1.1% for bioprostheses, with a frequency of occurrence over 15 years that was similar for both groups (86% for bioprostheses and 85% for mechanical valves), as observed in the present cohort. Kulik et al. [23] observed differences in their cohort in terms of bleeding events and found that fitting a mechanical prosthesis was an independent factor for bleeding events (p = 0.02), which was not observed in this study.

In the present sample, 4.8% of the patients presented with renal failure. In a cohort of dialysis patients, Umezu et al. [27] identified bleeding events in 29.7% of the cases, which was a much higher percentage than those found in the current sample. The authors also observed a higher incidence of bleeding events in patients with mechanical valve replacements compared with biological valve replacements, which was not seen in the present cohort.

### Reoperation

Most of the publications available in the literature demonstrated that the risk of reoperation begins to increase 10 years after valve replacement surgery, most likely due to prosthesis dysfunction. This risk increases progressively over time and decreases with increasing age [17, 19]. In the present study, a trend towards reoperation was observed after 10 years of follow-up (p = 0.073).

The factors related to reoperation found in the current study were male gender, prosthesis diameter and endocarditis.

In a randomized trial by Oxeham (Edinburgh Trial) [2], reoperation was more frequent in the group of patients who underwent bioprosthesis implantation compared to mechanical prosthesis implantation (p < 0.001). In a clinical trial by Hammermeister et al. [13, 14], there was no significant difference in the probability of reoperation between the two types of prostheses (p = 0.23). In addition, in a review study, Rahimtoola [9] concluded that the major disadvantage of using a bioprosthesis is the higher incidence of reoperation, which can result in a higher mortality rate.

Endocarditis was also a predictor for reoperation in a study by Yau et al. [24], with HR = 8.93; 95% CI: 1.16–68.7; p = 0.04.

The 1062-patient cohort followed by Ruel et al. [26] had rates of survival free from reoperation after replacement with mechanical valves of 96.4%, 94.8% and 94.2% for 10, 15 and 20 years after surgery, respectively, similar to the data from the present cohort. For bioprostheses, survival free from reoperation rates were 79.8%, 63.3% and 57.6% (p < 0.001). Advanced age was a protective factor against reoperation due to structural mitral bioprosthesis dysfunction (HR = 0.98; p ≤ 0.001), which can be attributed to reduced deterioration of the prosthesis in older patients.

Jamieson et al. [21] assessed over a 15 year period the performance of 959 biological valve prostheses implanted in 943 patients and 961 mechanical prostheses implanted in 839 patients in the mitral position. The reoperation rate per year for mechanical prostheses was 0.5 events per 100 patients, and for biological prostheses, it was 3.7 events per 100 patients. The predictors for reoperation were age (n = 240, mean 56.0 ± 12.9 years, HR = 0.98, 95% CI 0.97–0.99) and the type of prosthesis, where a frequency of 2.7% was found for mechanical valves (n = 26) and a frequency of 22.2% was found for bioprostheses (n = 214, p = 0.001, HR = 0.19; 95% CI: 0.13–0.29, p < 0.001).

Hammermeister et al. [14], Ruel et al. [19], Khan et al. [20], Jamienson and cols [21], as in this present study, in the first five years was the higher incidence from free of deaths, valve-related reoperation and bleeding.

### Study limitations

This is a retrospective and observational study, and it is subject to bias and confounding factors that cannot be measured. In addition, it was conducted in a single center and exhibited an insufficient sample size for identifying rare events.

## Conclusions

The choice of replacement valve type remains a decision that should be taken by physician and patient in conjunction and must be individualized, taking into consideration the risks of reoperation and of chronic anticoagulation and their consequences, plus the comorbidities, life expectancy and characteristics of the patient and their lifestyle, in order to increase life expectancy and quality of life. Studies with long-term follow-up that can help in this choice are therefore relevant.

Although the incidence of death and reoperation were significantly higher in patients who underwent valve replacement with a biological substitute, the findings in this cohort demonstrated that the type of prosthesis was not an independent predictor associated with any of the clinical outcomes assessed, death, bleeding events and reoperation, using multivariate regression analysis. The mortality data obtained in this study were in agreement with the current literature, with 5, 10, 15 and 20 year survival rates similar in the two groups. Some of the results found in this study, such as the predictors found in the multivariate analysis, were also reported in the literature, while others need to be further studied to clarify their importance.

## Abbreviations

ACC/AHA: American Heart Association
CI: Confidence interval
DATASUS: Departamento de Informática do Sistema Único de Saúde
HCPA: Hospital de Clínicas de Porto Alegre
HR: Hazard ratio
IBGE: Instituto Brasileiro de Geografia e Estatística
ICU: Intensive care unit
FIPE: Fundação de Incentivo à Pesquisa
NYHA: New York Heart Association
PEPI: Programs for Epidemiologists
STROBE: Strengthening the Reporting of Observational Studies in Epidemiology
SUS: Sistema Único de Saúde.
